# Poor motor skills in childhood predict bully victimization across the lifespan: A study of adults with Autism Spectrum Disorder

**DOI:** 10.1192/j.eurpsy.2023.843

**Published:** 2023-07-19

**Authors:** M. R. Glans

**Affiliations:** Faculty of Medicine and Health, University Health Care Research Centre, Örebro, Sweden

## Abstract

**Introduction:**

Children with autism spectrum disorder (ASD) are frequently clumsy and are more likely to be bullied compared to typically developing peers. The link between motor skills and bully victimization is poorly understood.

**Objectives:**

The aim of the current study was to evaluate the effect of poor motor skills in childhood on bully victimization from early life to adulthood in those with ASD.

**Methods:**

In this cross-sectional study, 182 adults diagnosed with ASD completed a questionnaire on their recollection of bully victimization at different stages of life and their performance in physical education (PE), as a proxy for motor skills, and academic skills at age 12. Prevalence rates of bully victimization (defined as bullied ≥twice monthly) were compared at different time periods between those with- and without a memory of poor motor skills by chi-square tests. Moreover, logistic regression evaluated the associations while adjusting for candidate covariates sex and academic skills.

**Results:**

Out of the total sample of 182 adults (mean age=33 years, 48% female), 50% reported below average performance in PE. Prevalence rates of bully victimization were more common in those categorized as having poor motor skills as compared to those without poor motor skills in all measured time periods; 72% vs 28% p=.001 in nursery school, 69% vs 31%, p>.001 at 7-9 years, 61% vs 39%, p=.001 at 10-12 years, 64% vs 36%, p>.001 at 13-15 years, 73% vs 27%, p=.005 at 16-18 years and 73% vs 27%, p=.009 in working life. The statistically significant associations seen in the prevalence comparisons remained in the logistic regression models.

**Conclusions:**

The present study adds to the small, but growing, body of literature supporting an association between poor motor skills and bully victimization amongst children and adolescents with ASD. Moreover, we showed that the effect of childhood clumsiness on bully victimization continues into adulthood. Possibly, poor motor skills and social deficits share the same biological pathways and contribute to the risk of being perceived as “different”, and consequently bullied, by peers.

**Disclosure of Interest:**

None Declared

